# CircWDR26 regulates endometrial carcinoma progression via miR-212-3p-mediated typing genes MSH2

**DOI:** 10.1186/s40001-022-00755-3

**Published:** 2022-07-27

**Authors:** Tao-Xiang Lei, De-Jian He, Jian Cao, Wang-Gui Lv

**Affiliations:** 1 Department of Gynecological Oncology Surgery, Chenzhou First People’s Hospital (The First Affiliated Hospital of Xiangnan University), No.849 Youth Avenue, Chenzhou, 423000 Hunan Province China; 2 Department of Emergency, Chenzhou First People’s Hospital (The First Affiliated Hospital of Xiangnan University), Chenzhou, 423000 Hunan Province China; 3 Medical Imaging Center, Chenzhou Fourth People’s Hospital, Chenzhou, 423000 Hunan Province China

**Keywords:** Endometrial carcinoma, CircWDR26, MiR-212-3p, MSH2

## Abstract

**Background:**

Circular RNAs (circRNA) are important in mediating tumor progression, but their roles in endometrial carcinoma (EC) are not fully understood yet. Many circRNAs are dysregulated and may contribute to EC progression. The functions of circWDR26 in EC remain unknown.

**Methods:**

The expression of circWDR26 in EC and adjacent normal tissues, and cell lines was determined by qPCR. The proliferation, apoptosis, migration, and invasion of EC cells was examined by CCK-8 assay, flow cytometry, wound healing assay and Transwell assay. The interaction between circWDR26, MSH2 and miR-212-3p was determined by luciferase assay. EC cells were inoculated into nude mice and tumor burden was determined by measuring tumor dimensions, size, and weight. The proliferative marker Ki67 in EC tissue was determined by immunohistochemistry.

**Results:**

The expression of circWDR26 in EC tissues or cell lines was higher than in the normal tissue or endometrial epithelial cells. Downregulation of circWDR26 resulted in attenuated proliferation, increased apoptosis, reduced migration and invasion of EC cells. Mechanistically, circWDR26 targeted and suppressed the expression of miR-212-3p. We further found that MSH2 was the novel target of miR-212-3p and was upregulated by circWDR26 via inhibiting miR-212-3p. In vivo experiment demonstrated that circWDR26 was essential for EC tumor growth.

**Conclusion:**

circWDR26 promoted EC progression by regulating miR-212-3p/MSH2 axis and provided novel insights into anti-cancer treatment.

## Introduction

Endometrial cancer (EC) is the most common gynecological cancer in high-income countries. More than 300,000 women were diagnosed with, and nearly 70,000 died of EC worldwide annually. Although the early stage ECs have relatively good prognosis, the high-grade ECs tend to recur [1]. Surgery is the initial treatment for EC, around 90% EC patients have some form of surgery [2]. Additionally, adjuvant chemotherapy increases survival in EC, especially those with late stage EC (Stage III/IV) [2–4]. However, the oncogenesis of EC is not fully understood yet, studies are still needed to elucidate the pathogenesis of EC and to identify new targets for EC treatment.

The functions of circular RNAs (circRNA) in cancers start to emerge in recent years. CircRNAs exert multiple roles in oncogenesis by promoting malignant cell growth and proliferation, increasing cellular invasiveness, sustaining cellular stemness, circumventing cellular senescence and death, and fostering drug resistance [5, 6]. CircRNAs also hold the potential to be novel biomarkers for cancer [7–9]. Research on circRNAs in EC is still limited. Chen et al. analyzed the transcriptome of circRNAs in EC and found significant difference from normal tissue [10]. The expression of circWDR26 is elevated in EC [11, 12], but its roles and underlying mechanisms is not well studied yet.

One of the action models of circRNAs is as a sponge for microRNAs (miRNA) [13, 14]. As a competing endogenous RNA (ceRNA), circRNA directly binds to miRNA(s) with complementary sequence, and thus suppresses its activities, including promoting mRNA degradation [15]. hsa_circRNA_0001776 suppresses growth and induces apoptosis of EC by targeting miR-182 [16]. Moreover, circRNA WHSC1 regulates miR-646 to facilitate EC progression [17]. MiRNAs play versatile roles in carcinogenesis [18]. MiR-449a and miR-145-5p can serve as prognostic biomarkers for EC [19]. MiR-1271 targets CDK1 to suppress EC cell growth and induce apoptosis [20]. MiR-212-3p suppresses tumor progression in multiple cancers by targeting different molecules. miR-212-3p attenuated hepatocellular carcinoma cell growth and invasion by inhibiting CTGF [21], while in ovarian cancer, miR-212-3p can target MAP3K3 [22]. However, the exact role of miR-212-3p in EC is not known. Exploiting bioinformatic tool, we found a potential binding site between circWDR26 and miR-212-3p, which is not reported yet, and the role of circWDR26/miR-212-3p interaction in EC oncogenesis remains unknown.

The most well-known function of miRNA is directly binding to mRNA and promotes its degradation, suppressing gene translation and function [23]. miR-212-3p directly suppresses the expression connective tissue growth factor interrupt the proliferation and invasion of hepatocellular carcinoma [21]. In addition, miR-212-3p attenuates group 3 medulloblastoma progression via targeting nuclear factor I/B [24]. Again, with the bioinformatic tool, we identified a potential binding site in *MSH2*, which are tightly associated with malignancy [25–28], for miR-212-3p. However, the role of MSH2 in EC is unexplored yet. Therefore, it would be interesting to elaborate the relationship between circWDR26, miR-212-3p, and MSH2 in EC development.

In our current study, we explored the role and underlying regulatory mechanism of circWDR26 in EC oncogenesis. We found that circWDR26 promoted the proliferation, survival, migration, and invasion of EC cells through miR-212-3p/MSH2 axis, providing novel insights in the treatment of EC.

## Materials and methods

### Tissue specimen collection

Tumors and adjacent normal tissues (*n* = 30) used in this study were obtained from The First Affiliated Hospital of Xiangnan University. The adjacent normal tissues were at least 5 cm away from tumor tissues. All patients did not receive any tumor-related treatment, including radiotherapy and chemotherapy before tissue collection. Tissues were snap-frozen and stored in − 80 °C. Lymph node metastasis was evaluated by diagnostic radiology and pathology. Moreover, the clinical characteristics of EC patients are provided in Table 2. The study was approved by The First Affiliated Hospital of Xiangnan University and performed in accordance with the provisions of the Declaration of Helsinki and Good Clinical Practice guidelines. Written informed consent was obtained from all patients. 

### Cell culture and transfection

Human endometrial cancer cell line RL95-2 (Catalog #CRL-1671, American Type Culture Collection, ATCC), Ishikawa (CL-0283, Procell Life Science & Technology Co., Ltd), HEC-1-A (Catalog #HTB-112, ATCC), and HEC-1B (Catalog #HTB-113, ATCC) were cultured in complete DMEM (10% FBS, 100 units/ml penicillin, and 100 µg/ml streptomycin) at 37 °C in an atmosphere containing 5% CO_2_.

Human endometrial epithelial cells (hEEC) were isolated from endometrial specimens as previously described [29]. The endometrial tissue was minced in Ca^2+^/Mg^2+^-free PBS (Catalog #SH30256.01, Hyclone) and digested by type I collagenase (Sigma) and hyaluronidase for 1 h. Digested tissue were filtered through a 40-µm cell strainer (Falcon). The flow-through was discarded and materials remained in the filter were washout and cultured in stromal cell medium containing 67.5% complete DMEM (Catalog #12430054, Gibco), 22.5% MCDB-105 (Catalog #M6395, Sigma) at 37 °C for 1 h. Collected medium and unattached cells were centrifuged, resuspended in KSFM (Catalog #17005042, Gibco), and cultured for further experiments.

sh-circWDR26, pc-circWDR26, miR-212-3p mimic/inhibitor or the corresponding control plasmid were obtained from GenePharma Co., Ltd (Shanghai, China), and co-transfected into cells by Lipofectamine 2000 (Catalog #11668-030, Invitrogen) following manufacture’s instruction. Cells were transfected with 2.5 µg plasmid and incubated for 48 h before further experiments. Transfection to cells in different plates was performed similarly with adjustment of the amount of all materials proportionally.

### Cell proliferation assay

Measurement of cell viability was performed by cell counting kit 8 (CCK-8) from Abcam (Catalog #ab228554) following manufacturer’s instruction. Briefly, 10^4^ cells were seeded in a 96-well plate with clear bottom. Ten microliter CCK-8 solution was added, and cells were incubated for 3 h at 37 °C free of light. The absorbance was measured at 460 nm.

### Colony formation assay

Cells were mixed with 1% agarose solution (Low gelling temperature agarose, Catalog #A9045, Sigma) and 2000 cells/well were seeded in 6-well plate pre-coated with 1% agarose. Cells were cultured for 2 weeks to allow colonies to form. Plates were fixed, followed by staining with 0.2% crystal violet solution (Catalog #C0775, Sigma). Excess staining was removed with 3 PBS washes, and cells were imaged with ChemiDoc scanner (BioRad). Cell colonies were counted by ImageJ software.

### Apoptosis assay

Apoptosis was determined by flow cytometry using Annexin V antibody and propidium iodide (PI). Single cell suspension was prepared by trypsinizing and ice-cold PBS washes. Cells were then incubated with Annexin V FITC (ANNEX300F, Bio-Rad) and ReadiDrop™ PI (1351101, Bio-Rad) for 1 h at 4 °C protect from light. Apoptotic cells were detected with Beckman Coulter Gallios Flow Cytometer and analyzed using FlowJo software (FlowJo, Ashland, USA).

### Wound healing assay

Seed 2 × 10^5^ cells into each well of 12-well culture plate, and culture for 18–24 h to reach 100% confluence. The cell layer was scraped with a 1-mm pipette tip, the detached cells washed out, and 1.5 mL fresh medium replenished. The cells were imaged using microscope as 0 h. Cells were incubated for another 48 h and imaged under microscope.

### Transwell assay

Cells were suspended in FBS-free media and seeded in top chambers of the Transwell plates, and 0.5 mL complete medium were added into the lower well as attractant. The inserts were taken out carefully 24 h later. The cells were fixed in 4% paraformaldehyde and stained with 1% crystal violet. The inserts were washed in PBS and the cells in the inner compartment of the inserts were gently removed. Cell number on the outer surface of the insert was counted and imaged under microscope.

### Western blotting

Protease inhibitor cocktail (cOmplete, Roche) containing RIPA buffer was used to extract cellular protein. A total of 20 µg proteins were loaded and separated by 10% SDS-PAGE and transferred onto polyvinylidene difluoride membrane. Blots was developed using enhanced chemiluminescence kits (Cat. #1705060S, BIO-RAD) as substrates and were visualized and captured by a ChemicDoc XRS system (Bio-Rad) and quantified by ImageJ software. Following antibodies were diluted at a ratio of 1:1000 for western blot experiment: MSH2 (Cat. #2017, Cell Signaling Technology), β-actin (Cat. #3700, Cell Signaling Technology).

### RNA isolation and real-time quantitative PCR (qPCR)

RNA was extracted from snap-frozen tissues and cells by RNeasy Mini Kit (Cat. No. 74104, QIAGEN) following manufacturer’s instruction. The quality and concentration of total RNA were checked by agarose gel electrophoresis and absorbance using NanoDrop 2000 spectrophotometer, respectively. cDNA was synthesized using High-Capacity cDNA Reverse Transcription Kit (Cat. #4368814, Applied Biosystems) from 1 µg of total RNA following manufacture’s instruction. The real-time qPCR was performed using SsoAdvanced Universal SYBR Green Supermix (Cat. #1725270, BIO-RAD) as following: 94 °C for 10 min, followed by 40 cycles of 94 °C for 15 s, 60 °C for 1 min. Glyceraldehyde 3-phosphate dehydrogenase (GAPDH) served as reference gene. The relative gene expression was calculated by 2^−ΔΔCt^. Primer information is provided in Table 1.

**Table 1:** Primers used in qRT-PCR

Gene	Primers (5′–3′)
circWDR26	F 5′- TGATGGCACTAAACTAGCAACAG -3′
	R 5′-TCCAATAGTTTAGATCGGGAAGC -3′
miR-212-3p	F 5’- CGCGAGATCAGAAGGTGATT -3′
	RT 5′- GTCGTATCCAGTGCAGGGTCCGAGGTATTCGCACTGGATACGACAGCCAC-3′
U6	F 5′- CTCGCTTCGGCAGCACA-3′
	R 5′- AACGCTTCACGAATTTGCGT-3′
MSH2	F 5′- TGGATCAGGTGGAAAACCAT-3′
	R 5′- ATCCAAACTGTGCACTGGAA-3′
GAPDH	F 5′- CCAGGTGGTCTCCTCTGA -3′
	R 5′- GCTGTAGCCAAATCGTTGT-3′

### Dual luciferase reporter gene assay

The binding sites between circWDR26, *TP53*, *LDLR, PUM2, LATS2, FOXN3* or *MSH2* and miR-212-3p were predicted by Starbase (https://starbase.sysu.edu.cn/). Predicted binding motifs and corresponding mutant (MUT) in above candidate genes were inserted into psiCHECK2 luciferase reporter vector (Promega, China). HEK293T cells were co-transfected miR-212-3p mimic with above luciferase reporter plasmids for 48 h. The relative ratio of Rluc/Luc was analyzed using the Dual-Luciferase reporter system (Promega, USA) as per the instructions.

### Xenograft EC mouse model

All animal experiments were performed in accordance with protocols approved by The First Affiliated Hospital of Xiangnan University. Balb/c female nude mice (6–8 weeks old) were obtained from Beijing Experimental Animal Center. Mice were maintained in a 12-h light/dark, 20–25 °C and 50–65 humidity specific pathogen-free animal facility with adequate food and water ad libitum and were acclimated for 1 week before tumor cell inoculation. A total of 1 × 10^6^ HEC-1-A cells expressing either sh-NC or sh-circWDR26 were injected subcutaneously. The dimensions of tumors were measured once a week. On day 35, mice were euthanized before they become moribund. Tumors were isolated for further analysis.

### Statistical analysis

Experiment was repeated for at least three times. Data were shown as mean ± SD. One-way ANOVA or Student’s *t*-test was used for statistical analysis with GraphPad Prism 8 software. *p* < 0.05 is considered statistically significant.

## Results

### The expression of circWDR26 is elevated in EC

**Fig. 1:**
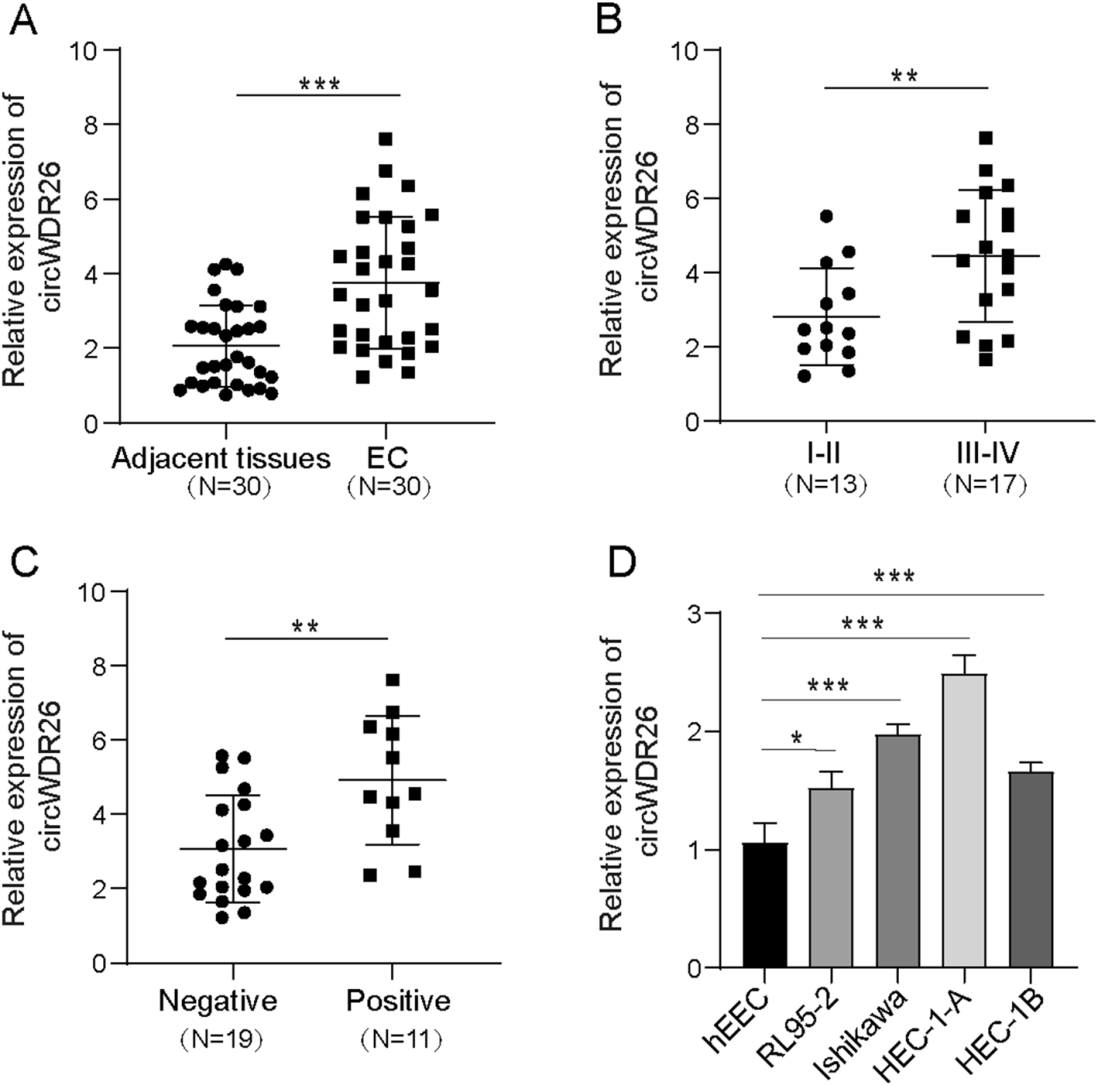
The expression of circWDR26 is elevated in EC. **A** The level of circWDR26 in normal tissue (NT) and EC tissue (EC) was determined by qPCR. **B** The level of circWDR26 in early stage (I–II) and late stage (III–IV) EC tissues was determined by qPCR. **C** The expression level of circWDR26 in tissues from patients with or without lymph node metastasis was determined by qPCR. **D** The expression of circWDR26 in hEEC and EC cell lines was determined by qPCR. *p < 0.05, **p < 0.01, ***p < 0.001

To investigate the expression pattern of circWDR26 in EC tissue, we compared the levels of circWDR26 in 30 normal tissues and 30 EC tissues. The expression of circWDR26 was dramatically upregulated in EC tissue (Fig. 1A). We next inquired whether the expression of circWDR26 changes along with tumor progression. The result indicated that the level of circWDR26 in EC tissue in late stages (Stage III–IV) were higher than in early stages (Stages I–II) (Fig. 1B). Level of circWDR26 was also positively correlated with metastasis of EC to lymph node. Tumor tissues from patients with lymphatic metastasis showed higher expression of circWDR26 than those without lymphatic metastasis (Fig. 1C). CircWDR26 expression in hEEC and EC cells were also examined, the level of circWDR26 was upregulated in all four EC cell lines compared with hEEC (Fig. 1D). Moreover, the clinicopathological characteristics, including age, tumor size, FIGO stage, differentiation, and lymphatic metastasis, of the 30 EC patients are provided in Table 2. These data demonstrated that the expression circWDR26 was upregulated in EC and was associated with tumor progression and metastasis, implying its role in EC pathogenesis.

**Table 2:** The clinical characteristics of EC patients

Characteristics	EC	
	Case (*N* = 30)	%
Age (year)		
≤ 50	14	46.67
> 50	16	53.33
Tumor size (cm)		
≤ 4	18	60.00
> 4	12	40.00
FIGO stage		
I–II	13	43.33
III–IV	17	56.67
Differentiation		
High and medium differentiation	16	53.33
Low and undifferentiation	14	46.67
Lymph nude metastasis		
Positive	11	36.67
Negative	19	63.33

*EC* endometrial carcinoma, *FIGO* Federation of Gynecology and Obstetrics

**Fig. 2:**
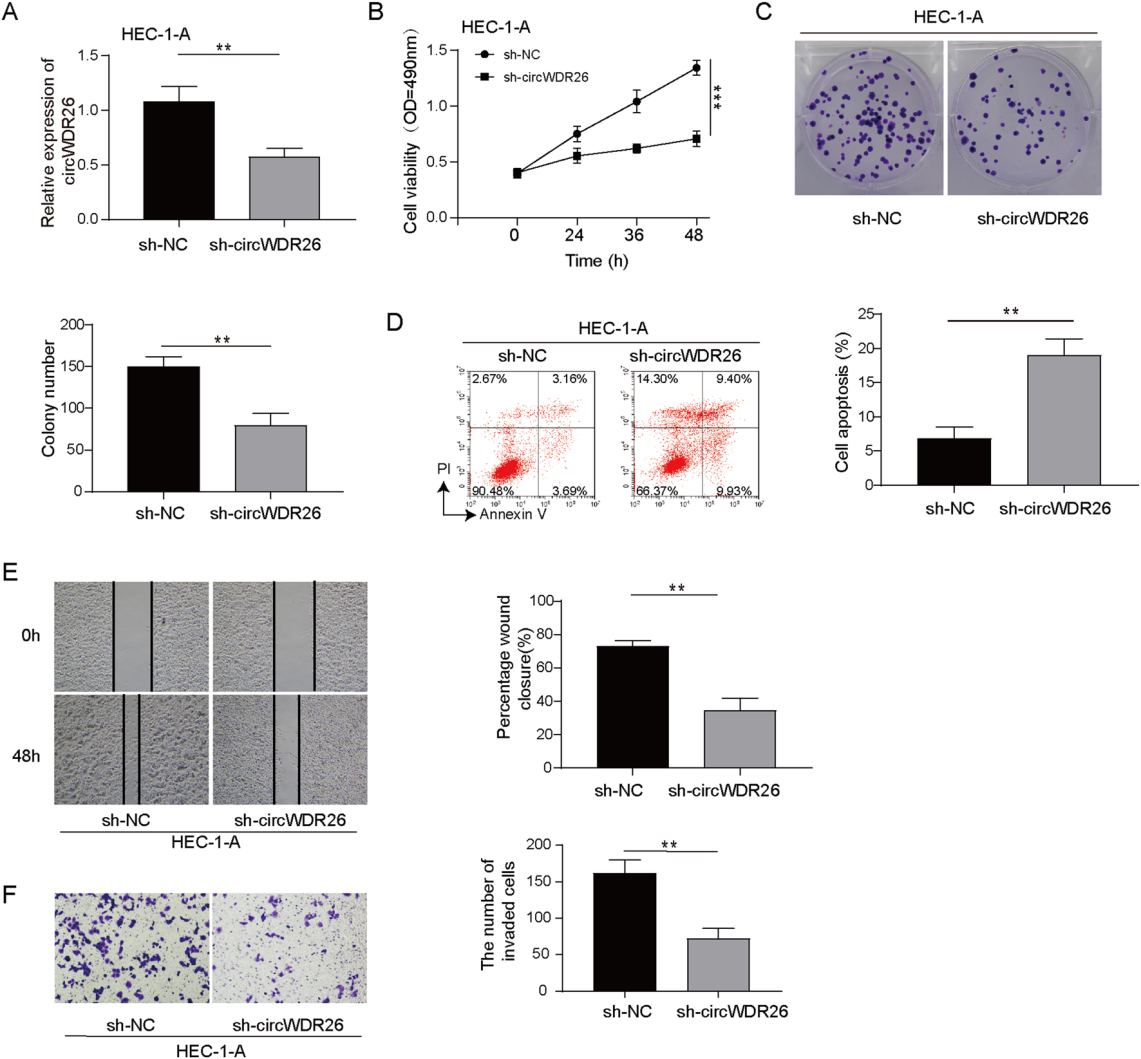
Loss of circWDR26 suppresses the proliferation, migration and invasion, and promotes apoptosis of EC cells. **A** Knockdown efficiency of circWDR26 was determined by qPCR. **B** CCK-8 assay for cell viability of sh-NC or sh-circWDR26 EC cells. **C** Colony formation of sh-NC or sh-circWDR26 EC cells. **D** Apoptosis was determined by flow cytometry stained with PI and Annexin V antibody. **E** Migration capacity of EC cells was determined by wound healing assay. **F** EC cell invasion was measured by transwell assay. **p < 0.01, ***p < 0.001

To examine its functions in EC, circWDR26 expression in EC cells were silenced by shRNA. As excepted, sh-circWDR26 effectively suppressed the expression of circWDR26 (Fig. 2A). The proliferation rate of EC cells was significantly attenuated by sh-circWDR26, compared with sh-NC (Fig. 2B). The clonogenic ability of EC cells was dramatically suppressed following circWDR26 downregulation (Fig. 2C). In addition, EC cells with lower circWDR26 expression exhibited higher rate of apoptosis (Fig. 2D). The migration capacity, as indicated in wound healing assay, of EC cells was decreased in sh-circWDR26 cells (Fig. 2E). Moreover, the invasion capacity of EC cells was also inhibited in cells expressing sh-circWDR26 (Fig. 2F). These data indicated that circWDR26 plays critical roles in proliferation, survival, migration, and invasion of EC cells.

### CircWDR26 acts as sponge of miR-212-3p

**Fig. 3:**
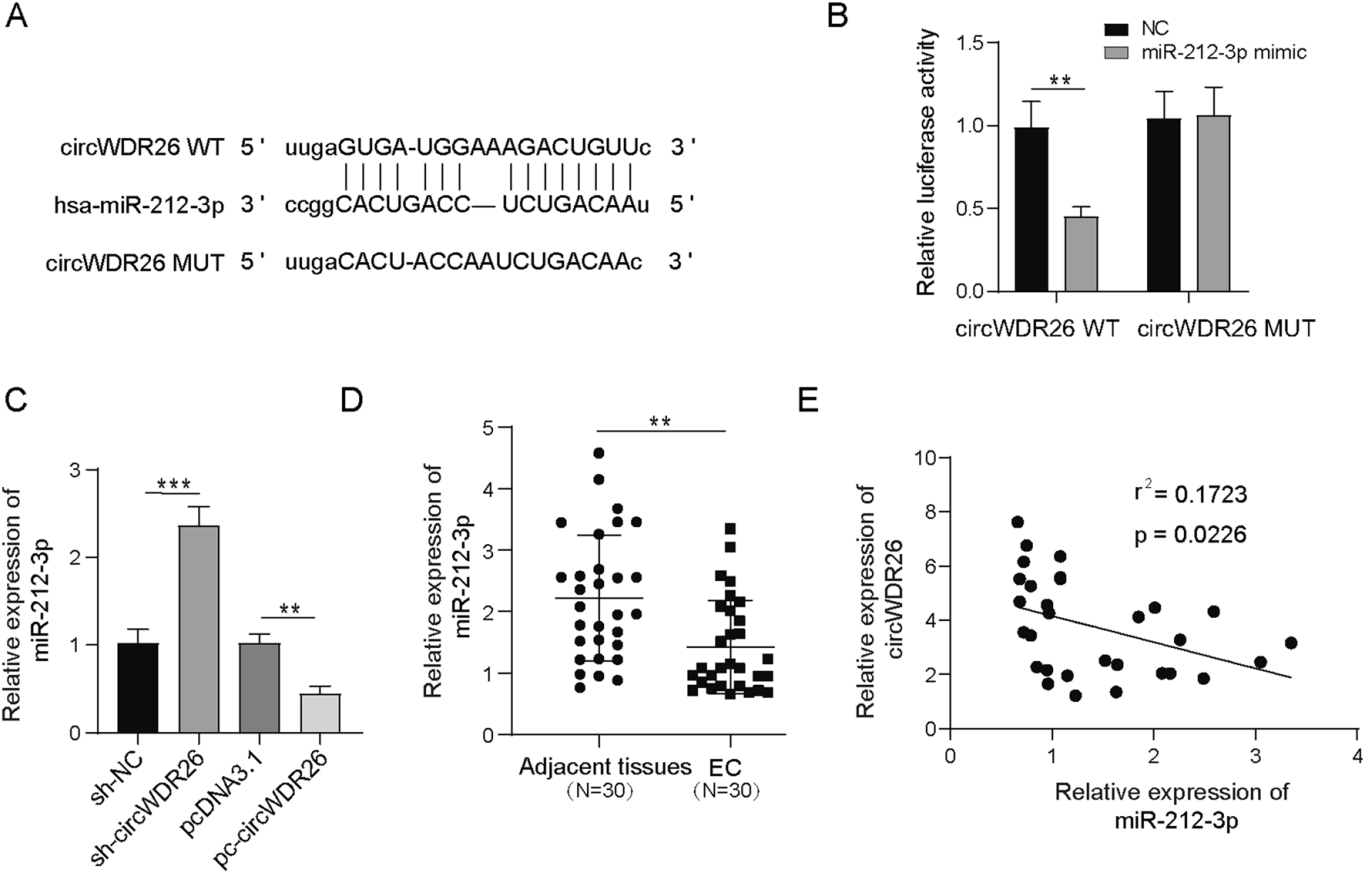
CircWDR26 acts as sponge of miR-212-3p. **A** Target prediction for circWDR26 using bioinformatic software Starbase. **B** Luciferase activity in cells co-transfected with miR-212-3p mimic and WT or MUT circWDR26. **C** The expression of miR-212-3p in cells knocked down or overexpressed circWDR26 was determined by qPCR. **D** The expression of miR-212-3p in normal and EC tissue was measured by qPCR. **E** The correlation of circWDR26 and miR-212-3p in EC tissue. *p < 0.05, **p < 0.01, ***p < 0.001

Next, we explored the possible targets of circWDR26 in EC cells. Bioinformatic analysis revealed that miR-212-3p contains potential binding site with circWDR26 (Fig. 3A). Luciferase activity in cells expressing WT circWDR26 was significantly suppressed by miR-212-3p mimic, while that in cells expressing MUT circWDR26 remained unchanged (Fig. 3B). In sh-circWDR26 expressing cells, the level of miR-212-3p was significantly increased, while overexpressing of circWDR26 reduced the expression of miR-212-3p (Fig. 3C). Consistently, significant reduction of miR-212-3p in EC tissue, compared with normal tissue, was observed (Fig. 3D). The expression level of circWDR26 was negatively associated with that of miR-212-3p (Fig. 3E). These findings suggested that miR-212-3p is a target of circWDR26 in EC.

### MiR-212-3p directly targets MSH2 in EC

**Fig. 4:**
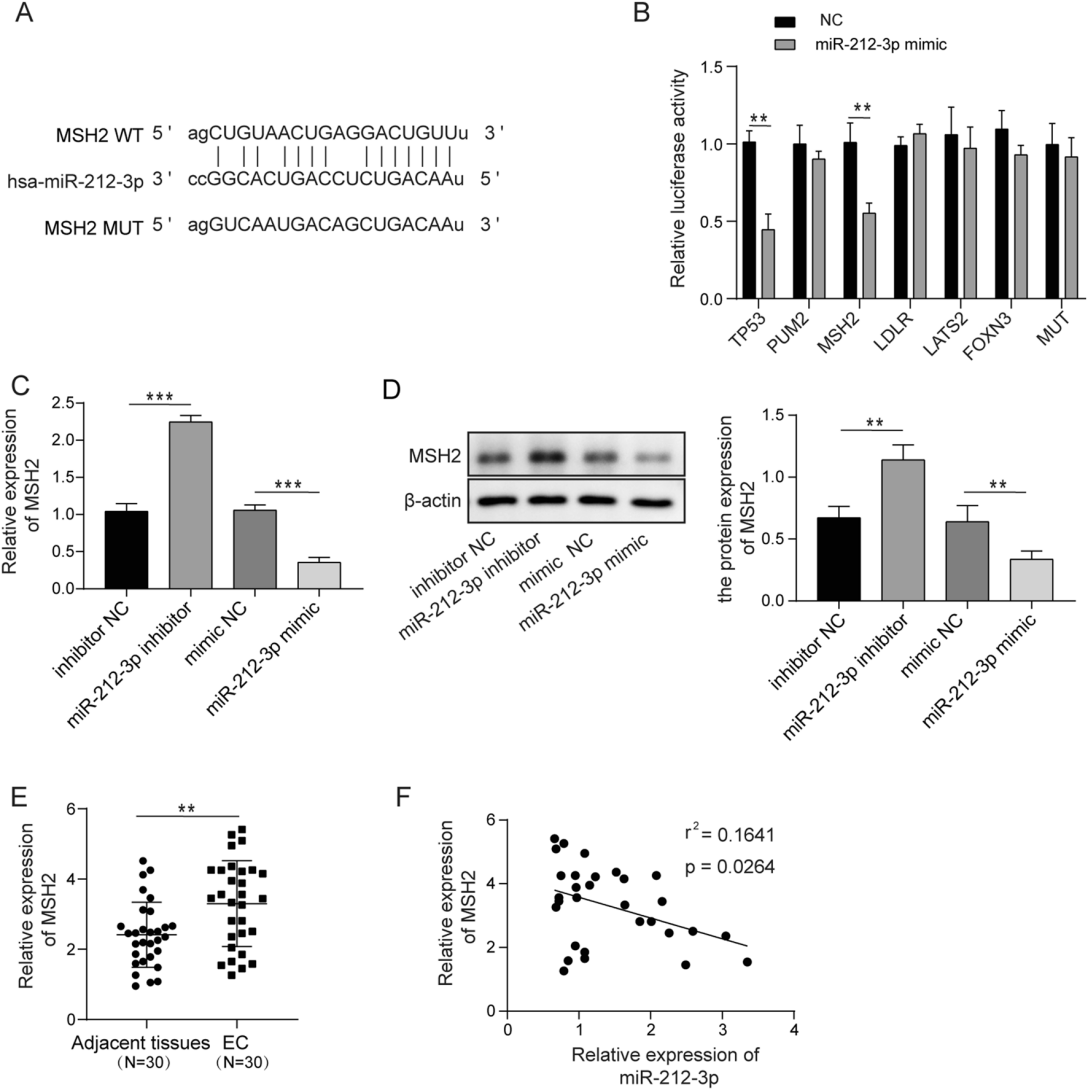
miR-212-3p directly targets TP53 and MSH2 in EC. **A** Target prediction for miR-212-3p using bioinformatic software Starbase. **B** Luciferase activity in cells co-transfected with miR-212-3p mimic and WT or MUT TP53, PUM2, MSH2, LDLR, LATS2, and FOXN3. **C**, **D** The expression of MSH2 in cells treated with miR-212-3p mimic or miR-212-3p inhibitor was determined by qPCR (**C**) or western blotting (**D**). **E** The expression of p53 MHS2 in normal and EC tissue was measured by qPCR. **F** The correlation between miR-212-3p and MSH2 expression in EC tissue. *p < 0.05, **p < 0.01, ***p < 0.001

Several potential targets (TP53, PUM2, MSH2, LDLR, LATS2, and FOXN3) of miR-212-3p were predicted by the Starbase software (Fig. 4A). Subsequently, we further examined if these candidate genes were regulated by miR-212-3p using dual-luciferase assays. Results revealed that miR-212-3p mimic reduced the luciferase activity of WT TP53 and MSH2, but there was no statistical differences in other candidate genes and MUT (Fig. 4B). Similarly, miR-212-3p inhibitor promoted the expression of MSH2, but miR-212-3p mimic decreased the levels of MSH2 (Fig. 4C). The protein levels of MSH2 were consistently upregulated by miR-212-3p inhibitor and downregulated by miR-212-3p mimic (Fig. 4D). Concomitant with reduced expression of miR-212-3p in EC, the expression of MSH2 was dramatically increased in EC tissue, compared with normal tissue (Fig. 4E), and MSH2 expression was negatively correlated with the expression of miR-212-3p (Fig. 4F). These results demonstrated that miR-212-3p negatively regulated MSH2 expression in EC.

### CircWDR26 promotes EC progression by regulating miR-212-3p/MSH2 axis

**Fig. 5:**
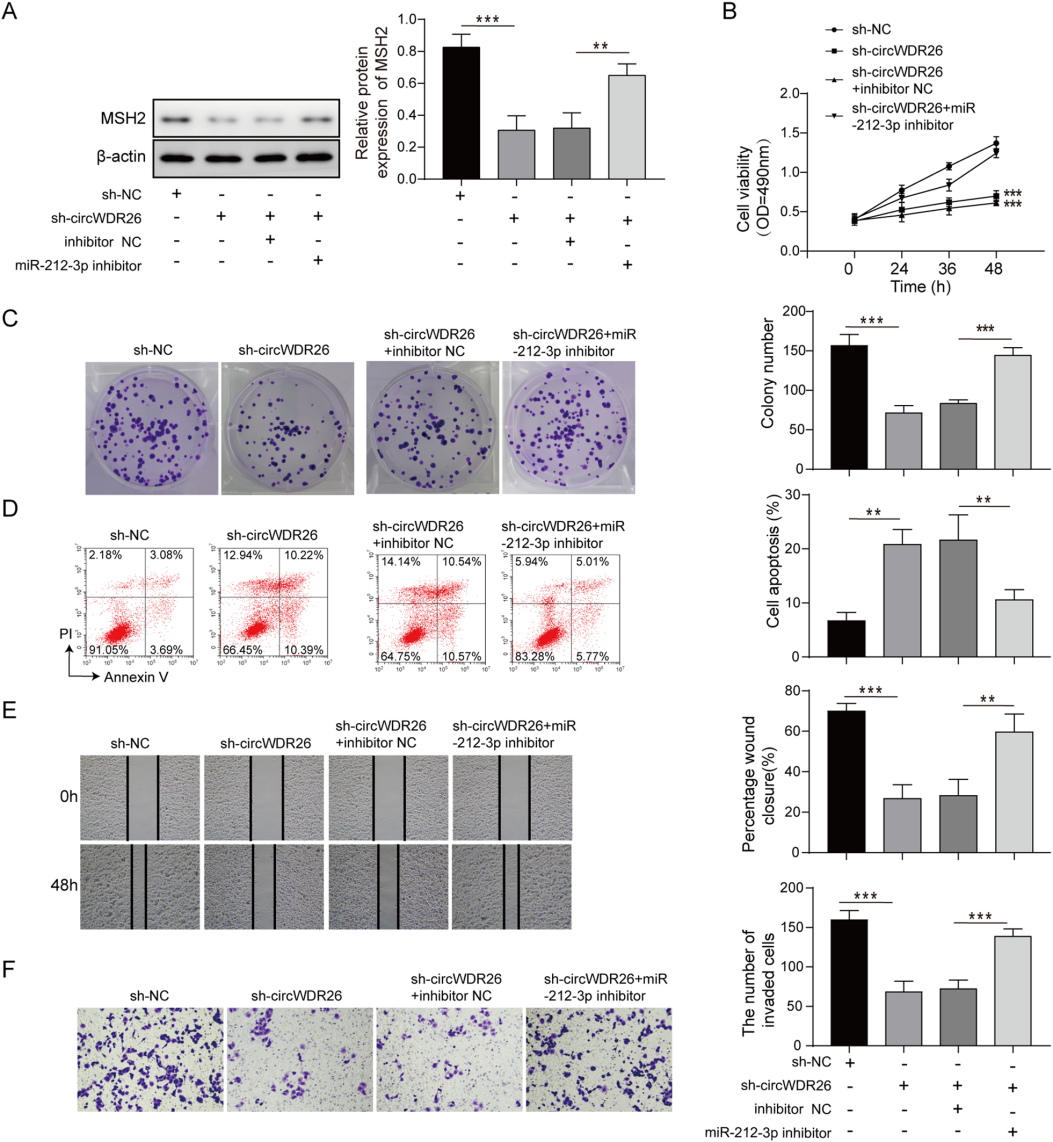
CircWDR26 promotes EC progression by regulating miR-212-3p/MSH2 axis. **A** The expression of MSH2 was determined by western blotting. **B** Cell viability was measured by CCK-8 assay. **C** Colony formation capacity of EC cells was determined in soft agar. **D** Apoptosis was determined by flow cytometry stained with PI and Annexin V antibody. **E** Migration capacity of EC cells was determined by wound healing assay. **F** EC cell invasion was measured by Transwell assay. *p < 0.05, **p < 0.01, ***p < 0.001

To investigate whether miR-212-3p/MSH2 axis is required for circWDR26-mediated EC progression, we first examined the expression of MSH2 following manipulation of circWDR26 and miR-212-3p. Knockdown of circWDR26 decreased the expression of MSH2, while additional miR-212-3p inhibitor reversed the suppression of MSH2 in sh-circWDR26 cells (Fig. 5A). Cell proliferation was attenuated in EC cells expressing sh-circWDR26, while miR-212-3p inhibitor offset the inhibitory effect of sh-circWDR26 (Fig. 5B). The suppressed clonogenic capacity of EC cells in sh-circWDR26 was also enhanced with the addition of miR-212-3p inhibitor (Fig. 5C). Moreover, loss of circWDR26 promoted apoptosis, but further inhibition of miR-212-3p in these cells prevented them from apoptosis (Fig. 5D). sh-circWDR26 reduced migration of EC cells, while miR-212-3p inhibitor enhanced their migratory activity (Fig. 5E). Similarly, invasiveness of EC cells was suppressed by sh-circWDR26, but miR-212-3p inhibitor attenuated the suppressive activity caused by loss of circWDR26 (Fig. 5F). Collectively, these data indicated that circWDR26 regulated the expression of MSH2 through miR-212-3p, and miR-212-3p was indispensable for its regulation of EC cell proliferation, apoptosis, migration, and invasion.

### Loss of circWDR26 attenuated EC tumor growth in vivo

**Fig. 6:**
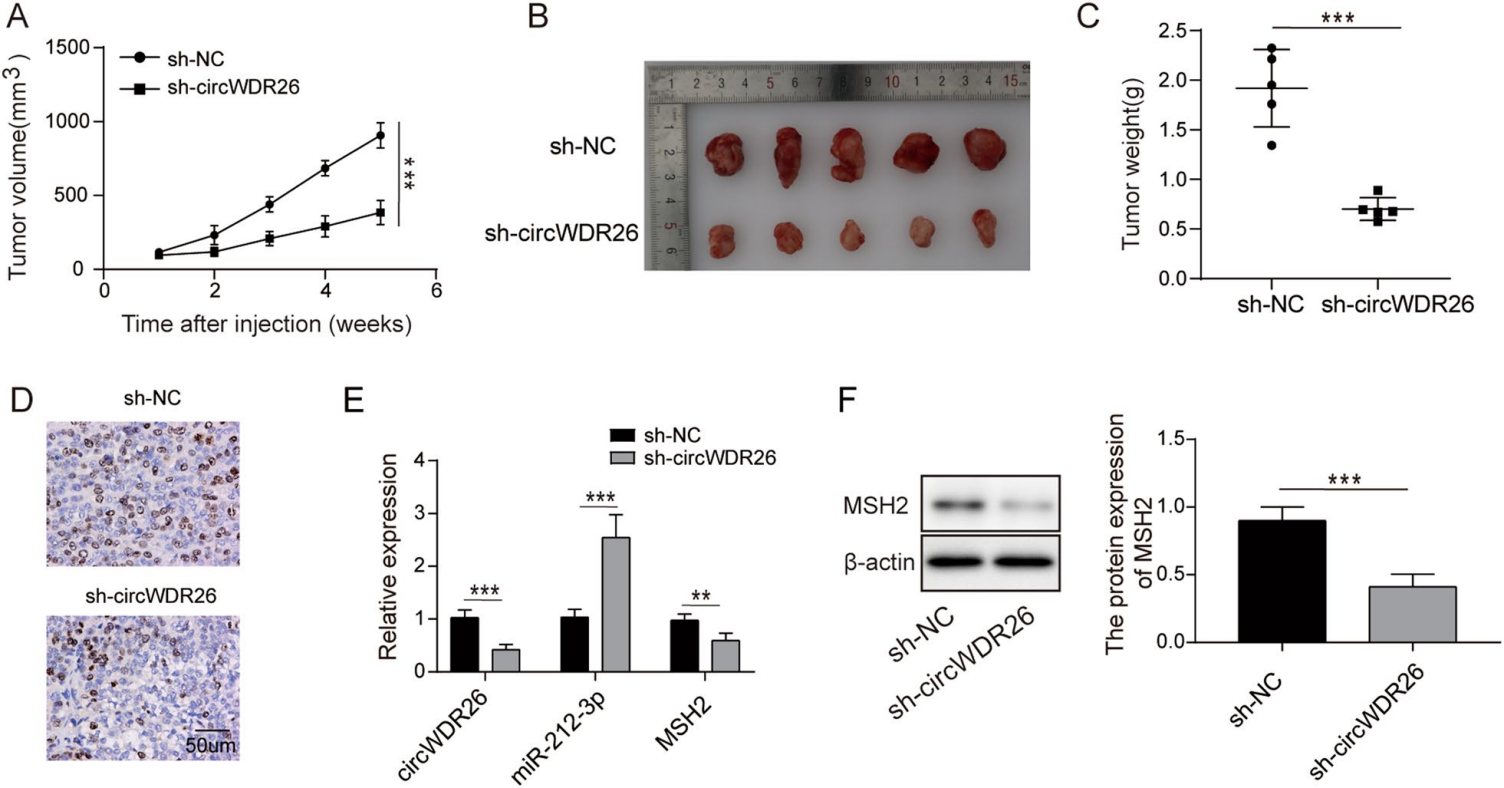
Loss of circWDR26 attenuated EC tumor growth in vivo. **A** Tumor dimensions were measured every week. **B** Tumor images of sh-NC or sh-circWDR26 group. **C** Weight of tumors. **D** Ki67 expression in tumor tissues was determined by immunohistochemistry. **E** Expression of circWDR26, miR-212-3p, and MSH2 in tumor tissues was determined by qPCR. **F** Protein levels of MSH2 in tumor tissues were examined by western blotting. *p < 0.05, **p < 0.01, ***p < 0.001

Our previous data showed important role of circWDR26 for EC cells, we next investigated if circWDR26 is essential for EC tumor growth in vivo. EC cells expressing control shRNA or circWDR26-targeting shRNA were inoculated into nude mice. The tumor volume was measured once a week. Sh-circWDR26 tumors had significant slower growth rate than the control tumors (Fig. 6A), which was consistent with the tumor size and weight (Fig. 6B and C) at the end point. Immunohistochemistry assay showed decreased level of proliferation marker Ki67 in sh-circWDR26 tumor (Fig. 6D). The expression of circWDR26 and its downstream effectors miR-212-3p, MSH2 in tumor tissues was examined by qPCR. Concomitant with reduced circWDR26 in sh-circWDR26 tumor, miR-212-3p expression was upregulated, while MSH2 was downregulated (Fig. 6E). Accordingly, the protein level of MSH2 were reduced significantly in sh-circWDR26 tumor tissues, when compared with sh-NC tumor tissues (Fig. 6F). Therefore, our data demonstrated that circWDR26 regulates EC tumor progression in vivo through a miR-212-3p/MSH2 axis.

## Discussion

EC remains a huge health concern as around 300,000 new cases were diagnosed annually and nearly 70,000 died from EC worldwide [1]. Metastasis and recurrence are common in patients with high-grade EC [2]. Plenty studies in recent years have revealed the versatile functions of circRNAs in the pathogenesis of EC [30, 31]. Among them, the role of circWDR26 in the pathogenesis of EC is still poorly understood. Our current study discovered novel roles of circWDR26 in promoting the development of EC through a miR-212-3p/MSH2 axis-dependent manner.

The important roles of circRNAs in cancers are emerging [5, 32, 33], but more researches are still required to understand their roles and underlying mechanisms which regulate tumor progression. WDR26 was upregulated in multiple cancers to promote tumor progression [30, 31]. Upregulated WDR26 may also lead to elevation of circWDR26. Previous studies have reported the roles of circWDR26 in promoting EC progression by activating Wnt/β-catenin pathway and IGF1R/PI3K/Akt pathways [11, 12]. Here we found circWDR26 promoted cultured EC cell proliferation, survival, migration and invasion. Given its important roles in EC, it is not surprising that the expression of circWDR26 was elevated in tumor. CircWDR26 expression in EC cells was upregulated and was elevated in EC tissues, and it was upregulated along with tumor progression, as circWDR26 in tissues from late stages were higher than that from early stages. The expression of circWDR26 showed association with lymph node metastasis of EC. Reduced expression of circWDR26 in EC cells dramatically suppressed tumor growth. Collectively our data demonstrated that circWDR26 has multiple functions in EC and is essential for EC progression.

Many studies have revealed the classical model of circRNA function as miRNA sponges [13, 14]. Here we found circWDR26 regulated the activity of its target miR-212-3p in a similar way. Using bioinformatic tools, we found circWDR26 contains potential binding site for miR-212-3p, which was further experimentally validated by dual-luciferase assay. The level of miR-212-3p was also downregulated after circWDR26 knockdown. Moreover, loss of circWDR26 attenuated EC progression and can be rescued by miR-212-3p inhibitor, further emphasizing the regulatory role of circWDR26 on miR-212-3p expression to promote EC progression. miR-212-3p exhibits inhibitory activity in proliferation and migration of human hepatocellular carcinoma [21]. The expression of miR-212-3p is downregulated in ovarian cancer cells and tumor tissues [34], and miR-212-3p suppressed the progression of high-grade serous ovarian cancer [22]. Although miR-212-3p showed broad tumor suppressor activities in various cancers, its role in EC has not been reported yet. MiRNA usually directly binds to its mRNA targets and leads to its degradation, downregulating the expression of target genes [35]. We identified multiple candidate genes (TP53, PUM2, MSH2, LDLR, LATS2, and FOXN3) that contain potential binding site for miR-212-3p. While TP53 and MSH2, among them, were suppressed by miR-212-3p mimic in dual-luciferase assay. Moreover, we found TP53 was upregulated in EC, which is consistent with previous report [36], demonstrating that overexpression of TP53 is related to malignancy. On the contrary, we found that TP53 could play the role of antitumor in subsequent functional experiments, which is consistent with a previous report [37]. Meanwhile, it is well known that TP53 exerts the tumor suppressor role in cancers. These data suggested that the role of TP53 is controversial in EC and requires further comprehensive investigation in the future. Therefore, we focused on the function of MSH2 in EC in our current study. The expression of MSH2 was directly downregulated or upregulated by miR-212-3p mimic and miR-212-3p inhibitor, respectively. Inhibition of miR-212-3p led to concomitant MSH2 upregulation and EC progression. Germline MSH2 mutation was associated with higher risks of ovarian and endometrial cancer [25, 27], and the expression of MSH2 is associated with poor prognosis of uterine corpus endometrial carcinoma with a hazard ratio of 1.56 [38]. These data demonstrated that miR-212-3p regulated EC progression by MSH2. Taken together, our current study reveals that circWDR26 directly regulates the miR-212-3p-p53/MSH2 axis to affect EC tumor progression. This will help us understand the mechanism of EC tumors and provide new insights for the future treatment of EC.

## Data Availability

All data generated or analyzed during this study are included in this published article.

## Abbreviations

EC: Endometrial cancer
CircRNA: Circular RNA
MiRNA: MicroRNA
WT: Wild type
MSH2: Muts homolog 2
